# Differential Expression of Inflammasome-Related Genes in Induced Pluripotent Stem-Cell-Derived Retinal Pigment Epithelial Cells with or without History of Age-Related Macular Degeneration

**DOI:** 10.3390/ijms22136800

**Published:** 2021-06-24

**Authors:** Maria Hytti, Eveliina Korhonen, Heidi Hongisto, Kai Kaarniranta, Heli Skottman, Anu Kauppinen

**Affiliations:** 1 Immuno-Ophthalmology, School of Pharmacy, University of Eastern Finland, 70210 Kuopio, Finland; eveliina.korhonen@uef.fi; 2 Department of Clinical Chemistry, HUSLAB, Helsinki University Hospital, 00029 Helsinki, Finland; 3 Faculty of Medicine and Health Technology, Tampere University, 33014 Tampere, Finland; heidi.m.hongisto@tuni.fi (H.H.); heli.skottman@tuni.fi (H.S.); 4 Ophthalmology, School of Medicine, University of Eastern Finland, 70210 Kuopio, Finland; kai.kaarniranta@uef.fi; 5 Department of Ophthalmology, Kuopio University Hospital, 70029 Kuopio, Finland

**Keywords:** induced pluripotent stem cells, retinal pigment epithelium, age-related macular degeneration, inflammation, inflammasomes

## Abstract

Inflammation is a key underlying factor of age-related macular degeneration (AMD) and inflammasome activation has been linked to disease development. Induced pluripotent stem-cell-derived retinal pigment epithelial cells (iPSC-RPE) are an attractive novel model system that can help to further elucidate disease pathways of this complex disease. Here, we analyzed the effect of dysfunctional protein clearance on inflammation and inflammasome activation in iPSC-RPE cells generated from a patient suffering from age-related macular degeneration (AMD) and an age-matched control. We primed iPSC-RPE cells with IL-1α and then inhibited both proteasomal degradation and autophagic clearance using MG-132 and bafilomycin A1, respectively, causing inflammasome activation. Subsequently, we determined cell viability, analyzed the expression levels of inflammasome-related genes using a PCR array, and measured the levels of pro-inflammatory cytokines IL-1β, IL-6, IL-8, and MCP-1 secreted into the medium. Cell treatments modified the expression of 48 inflammasome-related genes and increased the secretion of mature IL-1β, while reducing the levels of IL-6 and MCP-1. Interestingly, iPSC-RPE from an AMD donor secreted more IL-1β and expressed more Hsp90 prior to the inhibition of protein clearance, while MCP-1 and IL-6 were reduced at both protein and mRNA levels. Overall, our results suggest that cellular clearance mechanisms might already be dysfunctional, and the inflammasome activated, in cells with a disease origin.

## 1. Introduction

Age-related macular degeneration (AMD) is a complex, multifactorial disease and the leading cause for vision loss among the elderly in the Western world. It is estimated that by 2040, patient numbers will increase up to 26 million cases in Europe alone, while worldwide numbers of AMD may reach 288 million cases [1,2]. Even today, AMD places a considerable burden on the healthcare system and the pressure to find new treatment therapies for this currently incurable disease continues to increase [3].

Retinal pigment epithelial (RPE) cells play a key role in the formation and progression of AMD. In the healthy retina, RPE cells maintain the functionality of photoreceptors, providing them with nutrients from the choroid and removing photoreceptor waste products. They also support the blood–retinal barrier as well as the retinal pH, and are involved in both the visual cycle and the immune defense of the central retina [4,5]. RPE cell degeneration is a consequence of a complex interplay between increased inflammation, mitochondrial dysregulation, deficient protein clearance, and increased oxidative stress.

RPE cell death is the critical first step that leads to photoreceptor degeneration and the loss of central vision that is a characteristic of AMD [5,6,7,8]. Consequently, RPE cells are vital for the in vitro modeling of AMD disease mechanisms. Unfortunately, primary RPE cells that could be used to model AMD progression are available only in very limited numbers and are mostly from late stage donors, i.e., they are collected after the onset of irreversible damage to many RPE cellular processes [9]. Recent advances made with induced pluripotent stem cell (iPSC)-derived RPE (iPSC-RPE) cells could solve many of these problems. iPSC-RPE cells have successfully been used to model RPE degeneration in retinitis pigmentosa, Best disease, ciliopathy, LCHADD pigment retinopathy, type 2 diabetes, and AMD [9,10,11,12,13,14,15,16,17,18,19]. Previous studies on iPSC-RPE cells from AMD patients (hereafter referred to as AMD-RPE) and healthy controls (hereafter referred to as control-RPE) indicated that AMD-RPE cells displayed a reduced activity of the mitochondrial antioxidant enzyme superoxide dismutase 2 (SOD2) and had a higher susceptibility to oxidative stress [9,17,18,19]. They also showed decreased autophagy and complement dysregulation [9,17,18]. Previous studies using iPSC-RPE analyzed these cells’ responses to oxidative stressors, such as H_2_O_2_ or A2E [9,19]. Here we exposed iPSC-RPE cells from either an AMD patient or a healthy control subject to the proteasome inhibitor, MG-132 and the autophagy inhibitor bafilomycin A1. We have previously shown that the resulting experimental cellular clearance dysfunction leads to inflammasome activation in RPE cells and the secretion of pro-inflammatory cytokines [20,21,22].

There is a strong link between inflammation and the development of AMD. It is widely accepted that a chronic, low-level inflammation persists in the RPE cells of AMD patients, causing their dysfunction and eventual degeneration [5,23,24,25]. Known molecular stress factors related to AMD development, such as mitochondrial dysfunction, protein clearance malfunction, and oxidative stress can all contribute to, and be induced by, inflammatory processes in RPE cells. Drusen deposits that accumulate between RPE and Bruch’s membrane during the progression of AMD contain inflammatory proteins, and immune cell infiltration is known to be related to the pathological changes observed in AMD [5,25,26]. Elevated levels of some pro-inflammatory cytokines e.g., interleukin (IL)-1β, IL-6, tumor necrosis factor (TNF)-α, and IL-18 have been observed in AMD patients and have been proposed as biomarkers for the disease [5,25,27,28,29]. Increased levels of IL-1β protein and mRNA were found in the plasma and the retina of advanced AMD patients, respectively [27,28]. In addition, increased levels of both IL-1β and IL-18 were detected in the serum of AMD patients homozygous for the high risk CC allele of complement factor H (*CFH*) [27]. The maturation of IL-1β and IL-18 is facilitated by the activation of inflammasomes, multi-protein complexes that play an important role in the innate immune response. Inflammasome activation has been associated with AMD development and components of the NACHT, LRR, and PYD domains-containing protein 3 (NLRP3)-inflammasome have been detected in advanced AMD [5,25,28,30,31,32]. Here, we demonstrate that inflammasome activation occurs in response to dysregulated cellular clearance mechanisms in iPSC-RPE cells and that AMD-RPE cells show chronically increased production of IL-1β.

## 2. Results

### 2.1. Control and AMD-Patient-Derived iPSC-RPE Monolayers Showed Mature RPE Characteristics

**Figure 1:**
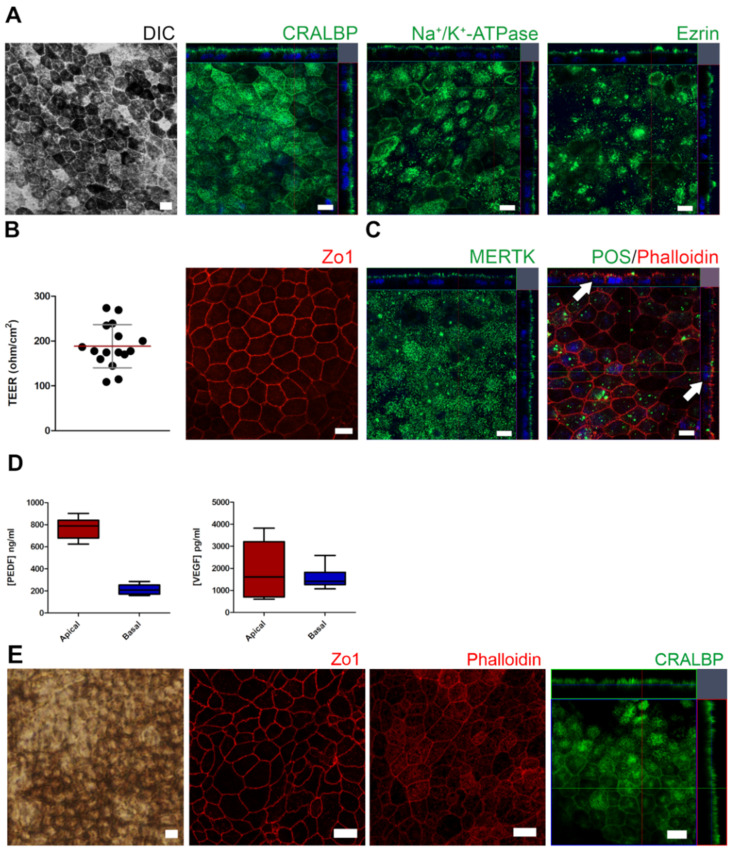
AMD-patient-derived iPSC-RPE monolayers showing mature RPE characteristics. (**A**) Differential interference contrast (DIC) image showing hexagonal RPE morphology with mosaic pigmentation and confocal z-stacks for immunofluorescence-labeled cells showing expression and polarized apical localization of RPE marker proteins. (**B**) Scatter-dot blot for transepithelial electrical resistance (TEER) measurements with standard deviation shown in gray and mean in red, n = 16 inserts from two separate differentiation experiments. Maximum intensity projection through the z-plane for tight junction protein zonula occludens-1 (Zo1). (**C**) Expression of phagocytosis ligand (MERTK) and internalized porcine photoreceptor outer segments (POS, arrows) after 2 h in vitro phagocytosis assay. Phalloidin for filamentous actin, Hoechst for nuclei, and scale bars 10 µm, valid for all images. (**D**) Secretion of pigment epithelial-derived growth factor (PEDF) was predominantly apical while vascular endothelial growth factor (VEGF) was secreted to both apical and basolateral insert compartments (n = 8 inserts from two separate differentiation experiments) represented as box plots showing growth factor secretion with whiskers for maximum and minimum. (**E**) Phase-contrast microscopy image showing hexagonal RPE morphology and pigmentation, as well as maximum intensity projection through the z-plane to show tight junction staining of ZO-1 and filamentous actin (phalloidin), as well as confocal z-stacks for immunofluorescence-labeled cells showing expression and polarized apical localization of RPE marker protein CRALBP in iPSC-RPE cells derived from a healthy control; scale bars 10 µm, valid for all images.

**Figure 2:**
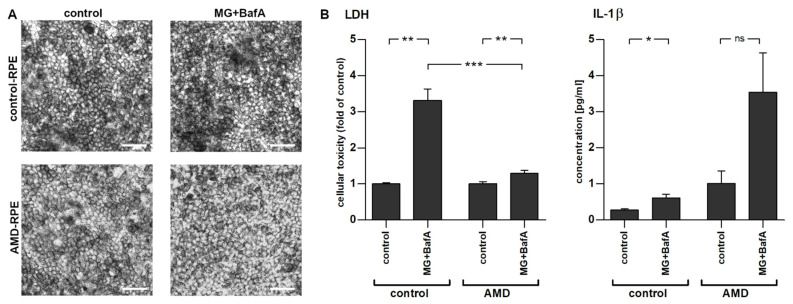
Effect of the exposure to 5 µM MG-132 and 50 nM bafilomycin A1 on IL-1α-primed iPSC-derived RPE cells from control and AMD subject. Representative images are shown, scale bars 50 µm. (**A**) Exposure to MG-132 and bafilomycin A1 did not induce phenotypical changes to iPSC-RPE cells. (**B**) MG-132 (MG) and bafilomycin A1 (BafA) caused an increase in LDH and IL-1β secretion. The data have been combined from three independent experiments with 2–6 parallel samples per group and are presented as mean +/− SEM. Ns, not statistically significant; *, *p* < 0.05; **, *p* < 0.01; ***, *p* < 0.001; Mann–Whitney U-test.

After reaching maturity, iPSC-derived RPE cells were characterized for morphology, RPE marker expression and polarity, tight epithelial barrier properties, and functional maturity (demonstrated as photoreceptor outer segment phagocytosis and secretion of growth factors). iPSC-RPE cells showed a normal RPE phenotype, with hexagonal RPE morphology and mosaic pigmentation irrespective of control- or AMD-origin. (Figure 1A,E and Figure 2A) Confocal microscopy analysis confirmed the expression and polarized apical localization of RPE marker proteins and the expression of tight junction protein zonula occludens-1 (Zo1) (Figure 1A,B,E). AMD-RPE cells were analyzed to confirm the secretion of pigment epithelial-derived growth factor (PEDF) and vascular endothelial growth factor (VEGF) (Figure 1D). The characterization confirmed that both control-RPE cells and AMD-RPE cells showed characteristics of mature RPE cells.

### 2.2. Inflammasome Activation in AMD- and Control-Patient-Derived iPSC-RPE Cell Lines

To stimulate inflammasome activation, iPSC-RPE cells were first primed with IL-1α and inflammasomes were then activated by inhibiting cellular clearance mechanisms using MG-132 (inhibition of the proteasome) and bafilomycin A1 (inhibition of autophagy). The conditions and concentrations had been previously tested and found to prevent protein clearance and induce inflammasomes in ARPE-19 cells and in embryonal stem-cell-derived RPE cells [22,33,34]. Activation of inflammasomes in iPSC-RPE cells significantly reduced cellular viability, as observed by an elevated release of lactate dehydrogenase (LDH). Interestingly, AMD-RPE cells were more resistant to the treatment and displayed a significantly smaller increase in LDH levels when compared to control-RPE cells (Figure 2B). Concurrently, the levels of IL-1β were significantly increased in control-RPE cells exposed to MG-132 and bafilomycin A1 when compared to vehicle-treated control-RPE cells (Figure 2B). AMD-RPE cells showed a 3.6-fold higher baseline secretion of IL-1β even before inflammasome activation, though this increase failed to reach statistical significance due to high variability between independent repetitions of the experiment (Figure 2; *p* = 0.17). MG-132 and bafilomycin A1 co-treatment increased IL-1β levels in AMD-RPE cells, but contrary to control-RPE cells, this increase remained statistically non-significant.

### 2.3. Inflammasome-Related Gene Expression in iPSC-Derived RPE Cells

**Figure 3:**
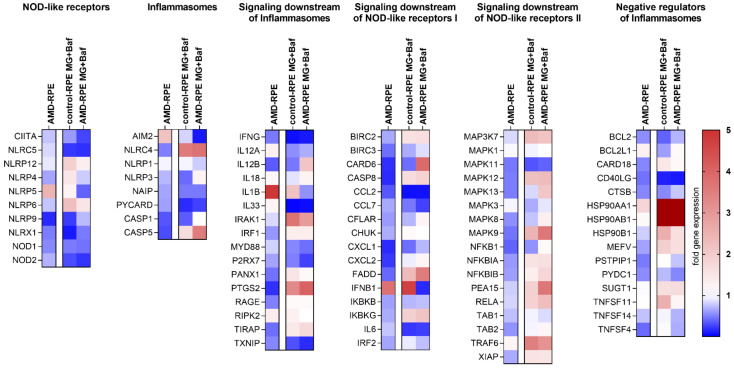
Expression levels of inflammasome-related genes determined by PCR Array. For each treatment group RNA from five samples extracted in three independent experiments was combined for analysis on a 96-well PCR array and data are presented as heatmaps. Data were analyzed using the 2^−ΔΔcT^ method, normalized to the housekeeping gene *B2M* and compared to the relevant control (i.e., vehicle-treated control-RPE for vehicle-treated AMD-RPE and MG + Baf-treated control-RPE, and vehicle-treated AMD-RPE for MG + Baf-treated AMD-RPE). MG, MG-132; Baf, bafilomycin A1.

The overall expression levels of inflammasome-related genes were examined in our AMD- and control-patient-derived iPSC-RPE cells by analyzing the mRNA levels in the cells using a human inflammasome polymerase chain reaction (PCR) array. Of the 89 genes studied, 26 were downregulated in AMD-RPE when compared to control-RPE cells, while 15 were upregulated (Figure 3 and Supplementary Materials, Figure S1). Notably, *IL1B* gene expression was upregulated by 4.89-fold, which could explain the higher baseline secretion of IL-1β observed in AMD-RPE cells (Supplementary Materials, Figure S1). Exposure to MG-132 and bafilomycin A1 caused predominantly comparable changes in the expression levels in AMD-RPE or control-RPE cells. Out of 48 genes that showed a change in gene expression in either or both cell lines after MG-132 and bafilomycin A1 exposure, the expression of 27 genes (56%) was changed equidirectionally. Interestingly, *IL1B* expression was upregulated after MG-132 and bafilomycin A1 exposure only in control-RPE cells, but not in AMD-RPEs, a result which might explain why the increase in IL-1β levels in the media of AMD-RPE cells after MG-132 and bafilomycin A1 exposure remained non-significant (Figure 2B). NLRP3 showed no changes in gene expression between control-RPE or AMD-RPE cells, while absent in melanoma 2 (AIM2) was 2.18-fold higher expressed in AMD-RPE cells when compared to control-RPE cells exposed to IL-1α priming and vehicle. Both NLRP3 and AIM2 can assemble inflammasomes and cause IL-1β secretion in RPE cells. *HSP90AA1* (heat shock protein 90) mRNA levels were the most strongly upregulated by exposure to MG-132 and bafilomycin A1 in both cell lines, with mRNA levels increasing by more than 15-fold compared to the situation in cells that were only primed with IL-1α (30.8-fold in control-RPE cells and 16.7-fold in AMD-RPE cells exposed to MG-132 and bafilomycin A1 when compared to their respective vehicle-treated controls). *HSP90AB1* and *HSP90B1* had also markedly increased expression levels after MG-132 and bafilomycin A1 exposure. Furthermore, the mRNA levels of *SUGT1*, which encodes ubiquitin ligase-associated protein suppressor of the G2 allele of SKP1 (SGT1), a protein that interacts with NLRP3 and Hsp90, were increased after the MG-132 and bafilomycin A1 exposure in both control-RPE and AMD-RPE. At the same time, gene expression levels of inflammatory cytokines chemokine (C-C motif) ligand 2 (*CCL2*, hereafter referred to as monocyte chemoattractant protein 1 (MCP-1)) and *IL6* were uniformly reduced (Figure 3). We chose to study the protein levels of Hsp90, MCP-1, and IL-6 in cell lysates or media to confirm the findings of the PCR Array.

### 2.4. The Levels of IL-6 and MCP-1, but Not IL-8, Were Reduced after MG-132 and Bafilomycin A1 Exposure in Control-RPE Cells

**Figure 4:**
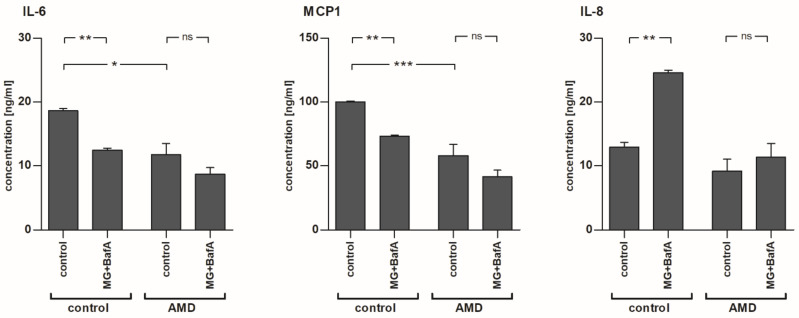
The levels of IL-6, MCP-1, and IL-8 in IL-1α-primed iPSC-derived RPE cells from a healthy control and an AMD patient with or without exposure to 5 µM MG-132 and 50 nM bafilomycin A1. The data have been combined from three independent experiments with 2–6 parallel samples per group and are presented as mean +/− SEM. Ns, not statistically significant; *, *p* < 0.05; **, *p* < 0.01; ***, *p* < 0.001; Mann–Whitney U-test.

In line with the mRNA expression data, we found that medium levels of IL-6 and MCP-1 were reduced in control-RPE cells after the exposure to MG-132 and bafilomycin A1 (Figure 4). Interestingly, the baseline secretion of IL-6 and MCP-1 to the medium was lower in IL-1α-primed AMD-RPE cells when compared to control-RPE cells. In fact, the levels of IL-6 and MCP-1 in MG-132 + bafilomycin A1-treated control-RPE cells were comparable to those of primed, vehicle-treated AMD-RPE cells (IL-6: 12.44 pg/mL and 11.75 pg/mL; MCP-1: 73.26 ng/mL and 57.92 ng/mL, for MG-132 + Baf-treated control-RPEs vs. vehicle-treated AMD-RPEs, respectively). Inhibition of proteasomes and autophagy in AMD-RPE cells did not significantly reduce the levels of IL-6 and MCP-1 further. Contrary to IL-6 and MCP-1, IL-8 levels were almost doubled in control-RPE cells after the exposure to MG-132 and bafilomycin A1, but this effect was not observed in AMD-RPE cells which appeared less reactive to protein clearance inhibition.

### 2.5. Increased Hsp90 mRNA Expression Levels Corresponded with Protein Levels

**Figure 5:**
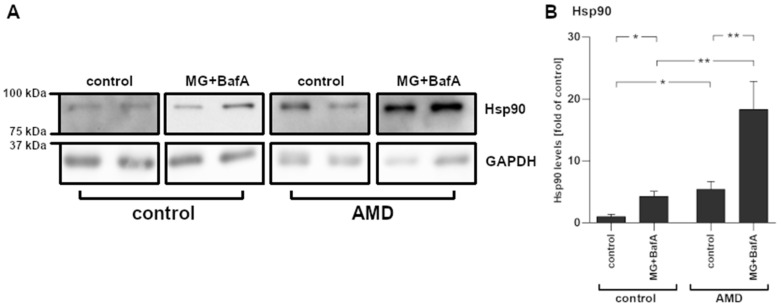
The levels of Hsp90 in IL-1α-primed iPSC-derived RPE cells from a healthy control and an AMD patient with and without exposure to 5 µM MG-132 and 50 nM bafilomycin A1 were measured using the Western blot technique. (**A**) Representative bands and (**B**) quantification of bands is shown. The data have been combined from three independent experiments with 2–6 parallel samples per group and are presented as mean +/− SEM. *, *p* < 0.05; **, *p* < 0.01; Mann–Whitney U-test.

The analysis of protein samples from cultured iPSC-derived RPE cells confirmed the presence of increased levels of Hsp90 after the exposure to MG-132 and bafilomycin A1 (Figure 5). Additionally, Hsp90 levels were significantly and more than 5-fold higher in AMD-RPE cells when compared to control-RPE cells. Similarly to the results observed in IL-1β, IL-6, and MCP-1, vehicle-treated AMD-RPE cells showed a protein expression pattern resembling that of control-RPE cells exposed to MG-132 and bafilomycin A1.

## 3. Discussion

Induced pluripotent stem cell (iPSC)-derived somatic cells from patients and healthy controls have provided researchers with the possibility to study complex diseases that are otherwise difficult to emulate in an in vitro setting. iPSC-derived cells from patients with complex diseases have been shown to exhibit disease-specific phenotypes in vitro and these cells could bridge the gap between clinical phenotype and molecular, cellular mechanisms [9,17,18,35,36,37]. Established culture protocols for iPSC-derived cells ensure that sufficient numbers of cells are available to conduct meaningful experiments, thereby creating a new, disease-specific platform for the identification of new mechanisms and revealing underlying pathways of disease [38].

RPE cells derived from iPSCs could help unravel the underlying cellular events of AMD. In the present study, cells derived from a control subject and an AMD patient were differentiated into RPE cells with appropriate RPE characteristics i.e., they displayed a pigmented and cobblestone-like morphology and expressed RPE-specific markers as well as exhibiting polarized secretion of PEDF and VEGF and a capability of undertaking in vitro phagocytosis of POS. After establishing the functionality and correct phenotype of our iPSC-RPE cells, we induced a dysfunctional waste clearance in IL-1α-primed RPE cells. The RPE is known to suffer an increasingly impaired intracellular clearance with aging which contributes to the development of AMD (reviewed in [7]). The compromised clearance of protein aggregates has also been associated with inflammation, and we have previously demonstrated its contribution to the activation of the NLRP3 inflammasome [20,22]. Inflammasomes are multi-protein complexes that are activated in response to a variety of extracellular or intracellular danger signals. The assembly of an active inflammasome results in the auto-activation of caspase-1, which subsequently cleaves the pro-inflammatory cytokines pro-IL-1β and pro-IL-18 into their mature and secreted forms [5]. Here we report that inhibition of the proteasome and of autophagy causes inflammasome activation, resulting in mature IL-1β secretion from iPSC-RPE. Furthermore, we found that AMD-RPE cells secreted more IL-1β than control-RPE cells at baseline, and that *IL1B* mRNA expression was increased in these cells, suggesting that inflammasome signaling is chronically activated in AMD-RPE cells. This result is in line with reports that IL-1β levels are increased in plasma samples of AMD patients carrying the high-risk CC allele of CFH and that *IL1B* mRNA levels are increased in the RPE of patients with advanced AMD [27,28]. In agreement with our findings, Hallam et al. have recently reported that iPSC-RPE cells from AMD patients secreted more IL-1β than those derived from healthy control subjects and that autophagy in these cells was dysfunctional [17].

Interestingly, AMD-patient-derived iPSC-RPE cells were more resistant to the cell death induced by the inhibition of proteasomes and autophagy than RPE cells derived from a healthy age-matched control. This is similar to reports by Ferrington et al. who found that primary RPE cells from AMD patients were more resistant to oxidation-induced cell death when compared to cells from healthy donors [39]. In contrast, Yang and colleagues described increased intracellular ROS levels in iPSC-derived RPE cells exposed to A2E and blue light while Golestaneh et al. found that AMD-RPE cells were more susceptible to H_2_O_2_-induced cell death [9,19]. Our model utilized very different stressors, i.e., inducing clearance malfunction and inflammasome activation. However, our results indicate that the chronic low-level inflammasome activation persisting in AMD-RPE cells could be cytoprotective against acute insults.

*HSP90AA1* was the gene displaying the largest expression change upon exposure to MG-132 and bafilomycin A1 in iPSC-RPE cells. The expression levels of *HSP90AB1* and *HSP90B1* were also markedly increased by the treatment. These genes encode for isoforms of the heat shock protein Hsp90, a multifunctional molecular chaperone that regulates the stability and the activation of several proteins related to signal transduction, protein trafficking, and immunity [22]. NLRP3, Hsp90, and SGT1, which also showed increased expression levels after MG-132 and bafilomycin A1 exposure, are part of an intracellular complex that protects NLRP3 from degradation [22]. Zuo et al. reported that the expression of inflammasome-related genes in mice suffering from subarachnoid hemorrhage was Hsp90-dependent [40]. They speculated that Hsp90 can assist in NLRP3 activation by stabilizing the P2X7 receptor [40]. Interestingly, increased Hsp90 levels have been observed in the RPE of AMD patients and the extent of the increase was directly proportional to AMD severity [41]. Correspondingly, we found that Hsp90 levels were higher in AMD-RPE cells when compared to control-RPE cells both at baseline and after MG-132 and bafilomycin A1 exposure. We have previously shown that Hsp90 inhibition can reduce NLRP3 inflammasome activation in RPE cells, and that this effect relied on active autophagy [22]. Further studies will need to be performed to test whether Hsp90 inhibition can alleviate the disease phenotype in AMD-RPE cells. Since it is known that Hsp90 can augment inflammasome activation through a number of pathways, the elevated Hsp90 levels in AMD-RPE further support the concept that a chronic inflammasome activation persists in these cells.

The PCR array analysis of inflammasome-related genes revealed that the *NLRP3* expression levels of AMD-derived iPSC-RPE cells did not differ from those of control-RPE cells. However, mRNA levels of *NLRP3* were very low, a finding that is in line with previous reports [42]. These low mRNA levels could have caused a false negative result in our analysis. We have previously shown that MG-132 and bafilomycin A1 exposure activated the NLRP3 inflammasome in ARPE-19, hESC-derived RPE, and primary RPE cells [22]. However, IL-1β maturation can also be facilitated by other inflammasomes. Interestingly, we found that *AIM2* levels were upregulated in AMD-RPE cells. AIM2 is a DNA-sensing receptor that can assemble an inflammasome and facilitate the maturation of IL-1β [5]. Recent reports by Kerur et al. suggest that the appearance of mitochondrial DNA in the cytosol is the critical stress signal leading to inflammasome activation and RPE cell death in a model of *Alu* RNA-driven retinal degeneration in mice [43]. Cytosolic mtDNA has been shown to activate the AIM2 inflammasome and cause the maturation and secretion of IL-1β in macrophages [44]. In line with these findings, we have recently observed that the AIM2 inflammasome is involved in antimycin A-induced mitochondrial stress in RPE cells (personal communication). Mitochondrial dysfunction has been observed in the RPE of AMD patients and has been linked to disease onset [45,46,47,48,49]. We postulate here that chronic activation of the AIM2 inflammasome is responsible for the increased secretion of IL-1β from AMD-RPE cells and that this might be linked to mitochondrial dysfunction, but further studies are needed to confirm this speculation.

PCR array data and the values of protein levels in cell lysates or the medium were in line not only for Hsp90 but also for the pro-inflammatory cytokines IL-6 and MCP-1. We found that mRNA expression and protein levels of both IL-6 and MCP-1 were decreased following the MG-132 and bafilomycin A1 exposure in iPSC-RPE cells derived from a healthy donor. Furthermore, the AMD phenotype also reduced MCP-1 and IL-6 levels to a similar degree. Interestingly, in AMD-RPE cells, MG-132 and bafilomycin A1 exposure did not further change the secretion of the cytokines. Similarly, IL-8 levels did not change significantly in AMD-RPE cells after the exposure to MG-132 and bafilomycin A1, although this treatment caused a significant increase in IL-8 secretion in control-RPE cells. We have previously shown that IL-1β secretion leads to a secondary elevation in IL-8 secretion from immortalized RPE cells exposed to MG-132 and bafilomycin A1 [20]. Taken together, the differences in responsiveness to proteasome and autophagy inhibition between the cells suggest a reduced reactivity to acute inflammatory stimuli in AMD-RPE cells. MCP-1, IL-8, and IL-6 have all been associated with the development of AMD. Mice lacking CCL2 (MCP-1) expression exhibit retinal degeneration similar to AMD [26,50,51]. Conversely, higher MCP-1 levels were found in the serum of aged individuals, as well as in the aqueous humor of patients suffering from advanced dry AMD [26,52]. Similarly, and in contrast to our findings with iPSC-RPE cells, increased IL-6 levels have been found in serum and ocular fluids of AMD patients [23,24]. IL-6 is known to be involved in neoangiogenesis and plays a role in choroidal neovascularization in advanced AMD [24,26]. IL-8 is a potent chemokine, attracting inflammatory cells that are involved in AMD disease progression, and genetic variations of the IL-8 gene have been found to be associated with AMD [53]. Interestingly, a reduction of IL-6 levels was found to protect retinal ganglion cells after optical nerve injury [54,55]. It is feasible that reduced expression of IL-6 and MCP-1 in AMD-RPEs might indicate activated survival mechanisms and stress responses, such as chronic inflammation, in these cells, corresponding to the increased survival of AMD-RPE cells seen here in our experiments after MG-132- and bafilomycin A1-induced stress.

It is noteworthy that most of the proteins we studied, i.e., IL-1β, IL-6, MCP-1, and Hsp90, behaved similarly both after exposure to MG-132 + bafilomycin A1 and under AMD conditions (Supplementary Materials, Figure S1B). Additionally, many of the differences in mRNA expression levels that we observed between healthy donor-derived iPSC-RPE and AMD-RPE cells, i.e., increased IL-1β, interferon β (IFN-β), and Hsp90 mirrored previously reported changes in the RPE with AMD progression [28,41,43,56]. This suggests that the impairments in protein and waste clearance that we induced by proteasome and autophagy inhibition mimicked a proteostasis dysregulation that was already ongoing in our AMD-RPE cells. This would also agree with the findings of Hallam et al., and Golestaneh et al., that autophagy was dysfunctional in AMD-derived iPSC-RPE cells and with the study by Cerniauskas et al. that reported dysfunctional lysosomal clearance in AMD-RPE cells [17,19,57]. A limitation of the results presented here is the limited number of patient and control samples and derived cell lines. However, our findings are in line with reports from different laboratories using iPSC-RPE cells generated from other patient and control cells, reporting the same findings (i.e., increased IL-1β secretion in AMD-RPE cells and reduced autophagy) [17,19,57]. Overall, our findings suggest that iPSC-RPE cells derived from an AMD patient show signs of a disease-specific phenotype which includes a chronic low-level inflammasome activation and possibly proteostasis dysregulation, which could explain the appearance of drusen in the retina of AMD patients.

## 4. Materials and Methods

### 4.1. Cell Culture

The Ethics Committee of the Kuopio University Hospital approved the study (42/2014), and the tenets of the Declaration of Helsinki were followed. All participants signed an informed consent form. AMD-iPSCs and healthy control iPSCs were generated from peripheral blood monocytes of one AMD patient and one age-matched control (Table 1) by the commercial service provider Glykos Finland Ltd (Helsinki, Finland). The iPSC-RPE cells were differentiated from these iPSCs as described previously [35]. Briefly, human iPSCs were cultured on Biolaminin 521 matrix (Biolamina, Sundbyberg, Sweden) in Essential 8 Flex Medium (Thermo Fisher Scientific, Waltham, MA, USA) at 37 °C in a cell incubator providing a humidified atmosphere enriched with 5% CO_2_. For the RPE differentiation, iPSCs were detached to a single cell suspension with TrypLE Select Enzyme (Tryple, Thermo Fisher Scientific, Waltham, MA, USA) and transferred to ultra-low attachment plates (Corning Inc., Corning, NY, USA) in KnockOut Dulbecco’s modified Eagle’s Medium (ko-DMEM) supplemented with 15% KnockOut Serum Replacement, 2 mM GlutaMAX, 0.1 mM 2-mercaptoethanol (all from Thermo Fisher Scientific, Waltham, MA, USA), 1% non-essential amino acids, and 50 U/mL penicillin–streptomycin (both from Lonza, Basel, Switzerland). Embryoid body (EB) formation was induced with overnight induction with 10 μM blebbistatin (Sigma-Aldrich, Saint Louis, MO, USA). For the following 2 days, EBs were allowed to undergo spontaneous differentiation and on day 4, the EBs were plated down to 0.75 μg/cm^2^ Biolaminin 521 matrix and 10 μg/cm^2^ human placental collagen type IV (col IV; Sigma-Aldrich, Saint Louis, MO, USA) coating. The medium was thereafter changed three to four times a week. After 30–45 days of differentiation, pigmented foci were selected with a scalpel, dissociated with Tryple, and replated to similarly coated culture wells (RPE passage 1). Forty-five days later the iPSC-RPE was again replated (RPE passage 2) and 9 days later the iPSC-RPE cells were frozen. For the cell characterizations, the RPE cells (passage 3) were plated into PET inserts with 1 µm pore size and a filtration area of 0.3 cm^2^ (Millipore, Billerica, MA, USA) coated with Matrigel (44 µg/cm^2^, Corning Inc., Corning, NY, USA) at a density of 7 × 10^4^ cells/insert and cultured for 7–9 weeks. The cells were examined for the characteristics of the mature RPE phenotype as described previously [35]. In the remaining experiments, RPE cells (passage 3) were plated on Biolaminin 521 matrix/col IV-coated 12-well plates, at a density of 4 × 10^5^ cells/well and cultured for 10 weeks. Once the cells had fully differentiated, they were exposed to 4 ng/mL IL-1α (R&D Systems, Abington, UK) for 24 h. Next, 5 µM MG-132 (Calbiochem, San Diego, CA, USA) was added and the cells were incubated for another 24 h before addition of 50 nM bafilomycin A1 (Merck KGaA, Darmstadt, Germany). One day (24 h) after bafilomycin A1 addition, medium samples, as well as RNA and protein samples, were collected.

### 4.2. Lactate Dehydrogenase (LDH) Assay

Cell viability was assessed using the commercial Cytotox^®^ non-radioactive cytotoxicity assay (Promega, Madison, WI, USA) following the manufacturer’s instructions. The LDH levels of different treatment groups were compared to those obtained from IL-1α-primed, vehicle-treated controls.

### 4.3. Enzyme-Linked Immunosorbent Assay (ELISA)

The levels of IL-1β, IL-6, and IL-8 were measured from medium samples using BD OptEIA™ ELISA kits (BD Biosciences, San Diego, CA, USA). Chemokine (C-C motif) ligand 2 (CCL2)/monocyte chemoattractant protein 1 (MCP-1) levels were determined using the eBioscience Human CCL2 (MCP-1) ELISA Ready-SET-Go!™ (eBioscience, San Diego, CA, USA). All ELISAs were performed according to the manufacturer’s instructions and our previously established protocols [21]. We have previously confirmed that the BD OptEIA™ human IL-1β ELISA kit measures mature IL-1β and not its inactive pro-form [21].

### 4.4. Polymerase Chain Reaction (PCR) Array

RNA and protein were extracted using the Nucleospin^®^ RNA/protein kit (Macherey-Nagel, Dueren, Germany). The RNA concentration in samples was determined using a NanoDrop^®^ spectrophotometer (Thermo Fisher Scientific, Waltham, MA, USA) at a wavelength of 260 nm and nucleic acid purity was assessed using the ratio of absorbance at 260 nm and 280 nm (A260/280). Equal amounts of RNA from five different samples of the same treatment groups across all three independent repetitions of the experiment were combined before reverse transcription using the RT^2^ First Strand cDNA kit (Qiagen Sciences, Germantown, MD, USA). Samples were then probed for inflammasome-related gene expression using the Real-Time qPCR method and the commercial Human Inflammasomes RT^2^ Profiler™ PCR Array (Qiagen Sciences, Germantown, MD, USA) run on an Applied Biosystems™ 7500 Real-Time PCR System (Thermo Fisher Scientific, Waltham, MA, USA). All PCR reactions were performed according to the manufacturer’s instructions. The threshold cycle was determined by setting the baseline above background noise levels and within the lower third of the linear phase of the amplification plot. The baseline was the same for each individual run. The c_T_ values, i.e., the cycle number in which gene amplification crossed the threshold, was determined by the PCR system and values were exported for further analysis using the SABiosciences PCR Array Data Analysis Template (SABiosciences.com/pcrarraydataanalysis.php (accessed on 21 August 2018). Melting curve analysis was performed after the RT-PCR run to confirm PCR specificity. All samples passed the quality control guidelines provided by the PCR array manufacturer. Gene expression data were normalized to the expression levels of the housekeeping gene β2-microglobulin (*B2M*). Gene expression changes in vehicle-treated AMD-RPE and MG-132 + bafilomycin A1-treated control-RPE were compared to vehicle-treated control-RPE, while MG-132 + bafilomycin A1-treated AMD-RPE data were compared to vehicle-treated AMD-RPE.

### 4.5. Western Blotting

The protein concentration in samples extracted with the Nucleospin RNA/protein kit was determined using the BCA Assay (Thermo Fisher Scientific, Waltham, MA, USA). Protein samples of 150 µg were separated on a 10% SDS-PAGE gel at 200 V for 2.5 h. Protein bands were blotted onto nitrocellulose membranes (Amersham, Piscatawy, NJ, USA) using wet-transfer at 17 V and left overnight. Successful transfer was confirmed using Ponceau S (Merck KGaA, Darmstadt, Germany) staining. Membranes were blocked in 3% milk in a phosphate-buffered saline (PBS) solution containing 0.3% Tween-20 (Merck KGaA, Darmstadt, Germany). The same buffer, without milk, was used for washes. After three washes each lasting 5 min, the membranes were probed with a heat shock protein 90 (Hsp90) primary antibody (ADI-SPA-835F, Enzo Lifesciences, Farmingdale, NY, USA, 1:5000 in 0.5% bovine serum albumin in wash buffer) for 2 h at room temperature. The membranes were washed and primary antibody binding was detected by incubating the membranes with a horseradish peroxidase (HRP)-conjugated secondary anti-rat antibody (NA934, GE Healthcare, Chicago, IL, USA; 1:20,000 in blocking buffer) for 1 h at room temperature. After the final set of washes, bound antibody was detected with the enhanced chemiluminescent (ECL) assay for horseradish peroxidase (Millipore, Billerica, MA, USA) on Super Rx medical X-ray film (Fuji Corporation, Tokyo, Japan). Glyceraldehyde 3-phosphate dehydrogenase (GAPDH) served as the loading control and was detected from the membranes using a 1:15,000 dilution of primary GAPDH antibody (ab-8245, Abcam, Cambridge, UK) in PBS containing 0.1% Tween-20 and a 1:12,000 dilution of HRP-linked anti-mouse IgG antibody (NA931, GE Healthcare, Chicago, IL, USA) in PBS with 0.1% Tween-20. Protein bands were quantified using the Image J software (U.S. National Institutes of Health, Bethesda, MD, USA; http://rsb.info.nih.gov/ij.

### 4.6. Statistical Analysis

With the exception of PCR array analysis, at least three independent repetitions were performed for each experiment. Results of all experiments were combined and are presented as mean +/− SEM. Statistically significant differences were determined using the Kruskal–Wallis test, followed by pairwise comparison of treatment groups using the Mann–Whitney U-test. Results were considered statistically significant at *p* < 0.05. All statistical analyses were performed using the GraphPad prism software (GraphPad Software Inc., San Diego, CA, USA).

## Figures and Tables

**Table 1:** Clinical information of iPSC-derived RPE cell lines.

iPS-RPE Line	Clinical Diagnosis	Donor Age (Gender)	History of Smoking	Additional Medication (Duration/Number)
AMD-RPE	AMD wet one eye, dry one eye	71 (M)	Yes, not current	Blood pressure medication (5 years) Anti-VEGF injections: 2
control-RPE	Control	73 (M)	No	Blood pressure medication anticoagulants (both 12 years)

## Data Availability

All data generated for this study is available from the corresponding authors upon reasonable request.

## Supplementary Materials

The following are available online at https://www.mdpi.com/article/10.3390/ijms22136800/s1.
