# Supplementary material for: Differential Expression of Inflammasome-Related Genes in Induced Pluripotent Stem-Cell-Derived Retinal Pigment Epithelial Cells with or without History of Age-Related Macular Degeneration
Source: Int J Mol Sci. 2021 Jun 24;22(13):6800. doi: 10.3390/ijms22136800 (PMC8268331; doi:10.3390/ijms22136800)
Supplement: Supplementary file 1 [file ijms-22-06800-s001.zip › ijms-1249556-supplementary.pdf]

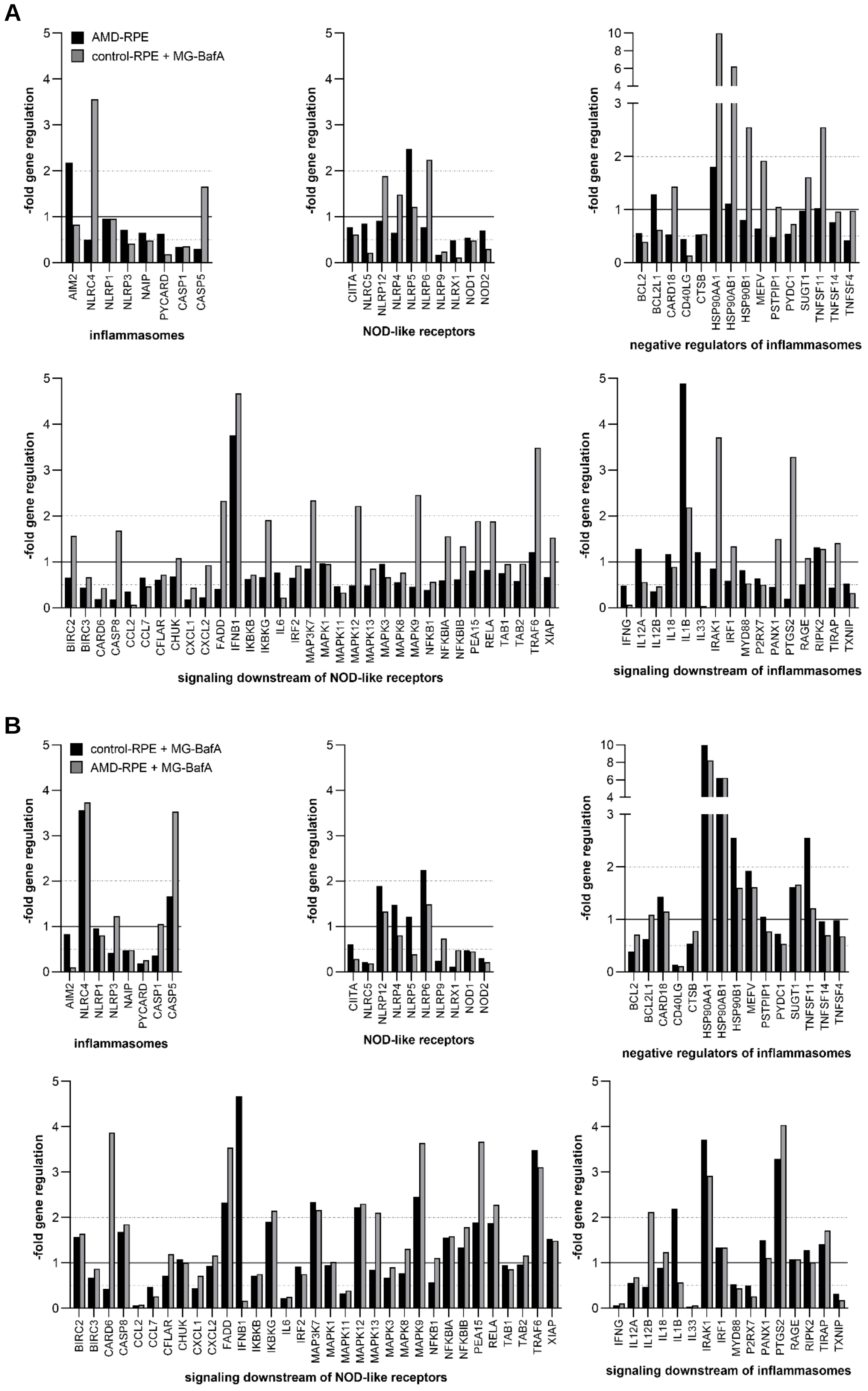
A
AMD-RPE
control-RPE + MG-BafA
-fold gene regulation
AIM2 NLRC4 NLRP1 NLRP3 NAIP PYCARD CASP1 CASP5
inflammasomes
CIITA NLRC5 NLRP12 NLRP4 NLRP5 NLRP6 NLRP9 NLRX1 NOD1 NOD2
NOD-like receptors
BCL2 BCL2L1 CARD18 CD40LG CTSB HSP90AA1 HSP90AB1 HSP90B1 MEFV PSTPIP1 PYDC1 SUGT1 TNFSF11 TNFSF14 TNFSF4
negative regulators of inflammasomes
BIRC2 BIRC3 CARD6 CASP8 CCL2 CCL7 CFLAR CHUK CXCL1 CXCL2 FADD IFNB1 IKBKB IKBKG IL6 IRF2 MAP3K7 MAPK1 MAPK11 MAPK12 MAPK13 MAPK3 MAPK8 MAPK9 NFKB1 NFKBIA NFKBIB PEA15 RELA TAB1 TAB2 TRAF6 XIAP
signaling downstream of NOD-like receptors
IFNG IL12A IL12B IL18 IL1B IL33 IRAK1 IRF1 MYD88 P2RX7 PANX1 PTGS2 RAGE RIPK2 TIRAP TXNIP
signaling downstream of inflammasomes
B
control-RPE + MG-BafA
AMD-RPE + MG-BafA
-fold gene regulation
AIM2 NLRC4 NLRP1 NLRP3 NAIP PYCARD CASP1 CASP5
inflammasomes
CIITA NLRC5 NLRP12 NLRP4 NLRP5 NLRP6 NLRP9 NLRX1 NOD1 NOD2
NOD-like receptors
BCL2 BCL2L1 CARD18 CD40LG CTSB HSP90AA1 HSP90AB1 HSP90B1 MEFV PSTPIP1 PYDC1 SUGT1 TNFSF11 TNFSF14 TNFSF4
negative regulators of inflammasomes
BIRC2 BIRC3 CARD6 CASP8 CCL2 CCL7 CFLAR CHUK CXCL1 CXCL2 FADD IFNB1 IKBKB IKBKG IL6 IRF2 MAP3K7 MAPK1 MAPK11 MAPK12 MAPK13 MAPK3 MAPK8 MAPK9 NFKB1 NFKBIA NFKBIB PEA15 RELA TAB1 TAB2 TRAF6 XIAP
signaling downstream of NOD-like receptors
IFNG IL12A IL12B IL18 IL1B IL33 IRAK1 IRF1 MYD88 P2RX7 PANX1 PTGS2 RAGE RIPK2 TIRAP TXNIP
signaling downstream of inflammasomes

**Supplementary Figure S1**: Expression levels of inflammasome-related genes determined by PCR Array. RNA from 5 samples extracted in three independent experiments were combined for analysis on 96-well PCR array and data are presented in a bar chart. A) MG-132 + bafilomycin A1-treated control-RPEs and IL-1$\alpha$-primed AMD-RPE cells are presented relative to IL-1a primed control RPE-cells (= 1). B) MG-132 + bafilomycin A1 treated control-RPE cells and MG-132 + bafilomycin A1 treated AMD-RPE cells are presented relative to their respective, corresponding IL-1a primed controls (= 1). Genes are grouped into groups according to the instructions provided by the PCR array manufacturer.
